# Operative lung cancer patients’ knowledge of pulmonary rehabilitation

**DOI:** 10.3389/fphys.2026.1707620

**Published:** 2026-02-19

**Authors:** Xiaowei Mao, Fang Hu, Jin Peng, Feng Pan, Jingjing Yan, Liyan Jiang

**Affiliations:** 1 Department of Pulmonary and Critical Care Medicine, Regional Medical Center for National Institute of Respiratory Diseases, Sir Run Run Shaw Hospital, School of Medicine, Zhejiang University, Hangzhou, China; 2 Pulmonary and Critical Care Medicine, Shanghai Jiao Tong University, Shanghai Chest Hospital, Shanghai, China; 3 Department of Thoracic Medical Oncology, Cancer Center, Hangzhou, Zhejiang, China; 4 Department of Respiratory and Critical Care Medicine, Hebei Petrochina Central Hospital, Langfang, China

**Keywords:** influence factors, knowledge, lung cancer, pulmonary rehabilitation, surgery, survey

## Abstract

**Introduction:**

We conducted this survey to explore what operative lung cancer patients knew about pulmonary rehabilitation and the factors that influence it.

**Methods:**

Between 1 January 2018 and 31 December 2020, patients who received thoracic surgery were enrolled in this study. We used a three-part questionnaire to collect the clinical features and knowledge of pulmonary rehabilitation.

**Results:**

A total of 93 patients were enrolled in this study. Most patients were female, ≤60 years old, had normal pulmonary function, and had been diagnosed with non-small-cell lung cancer. Univariate analysis revealed that patients with abnormal pulmonary ventilatory function, higher preoperative COPD assessment test (CAT) scores, higher CAT differences, and higher mMRC differences showed a higher awareness of pulmonary rehabilitation (p = 0.043, 0.029, 0.178, and 0.003, respectively). Multivariate analysis suggested that preoperative CAT score (p = 0.01) and mMRC difference (p = 0.001) were the factors associated with awareness of pulmonary rehabilitation.

**Conclusion:**

Many factors may influence the patients’ knowledge of pulmonary rehabilitation. We found that a higher preoperative CAT score and a larger mMRC difference were factors associated with awareness of pulmonary rehabilitation. However, assistance should also be provided to patients who do not fall into these categories, as they may lack knowledge of pulmonary rehabilitation.

## 1 Introduction

Lung cancer is the most frequently diagnosed cancer globally, responsible for almost 2.5 million new cases in 2022 (12.4% of all newly diagnosed cancers). Lung cancer is the leading cause of cancer death, with an estimated 1.8 million deaths (18.7% of all cancer deaths) (Bray et al., 2024). In China, lung cancer and chronic obstructive pulmonary disease were the third and fourth leading causes of years of life lost in 2017 (Zhou et al., 2019). In recent years, nanoscale imaging tools have assisted in lung cancer diagnosis and detection (Feng et al., 2024; Marcuello et al., 2025), which helps to detect more early-stage lung cancer.

Surgery is a valuable treatment for early-stage lung cancer patients within a multidisciplinary team (Bou-Samra and Singhal, 2024). Lung cancer patients are usually older, have a history of smoking, and suffer from cardiovascular or respiratory comorbidities (Gullón et al., 2012; Deng et al., 2020). These characteristics increase the risk of postoperative pulmonary complications (PPCs) (Ahuja et al., 2025). PPCs are regarded as the main causes of prolonged length of hospital stay, increased hospitalization costs, and poor quality of life.

Pulmonary rehabilitation is a meaningful intervention in the management of chronic obstructive pulmonary disease or other chronic respiratory diseases. In 2015, the American Thoracic Society/European Respiratory Society defined pulmonary rehabilitation as a well-designed pulmonary rehabilitation program that includes exercise training, pharmacotherapy, smoking cessation, nutritional support, behavior change, and health education (Rochester et al., 2015; Spruitm et al., 2013; Mao et al., 2021). Several studies have reported a shorter length of hospital stay, improved exercise tolerance, and a decreased rate of PPCs after pulmonary rehabilitation (Mao et al., 2021; Chen et al., 2022; Cruz Mosquera et al., 2024; Guo et al., 2025). Systematic analyses also confirm the clinical significance of pulmonary rehabilitation during the perioperative period (Mao et al., 2021; Chen et al., 2022; Cruz Mosquera et al., 2024; Guo et al., 2025).

Pulmonary rehabilitation is usually conducted by physicians, rehabilitation therapists, nutritionists, and nurses. The cognition of pulmonary rehabilitation among physicians or nurses has been reported previously by our research group (Zhang et al., 2021; Pan et al., 2024), but patient knowledge of pulmonary rehabilitation has been less frequently reported. We conducted this survey to explore the patients’ knowledge of pulmonary rehabilitation and the factors that influence it.

## 2 Methods

### 2.1 Patient selection

This survey was conducted between 1 January 2018 and 31 December 2020. This research was conducted in the inpatient and outpatient departments simultaneously. Patients who received thoracic surgery were enrolled in this study. Those who had previously received thoracic surgery or received bullae resection simultaneously were excluded. Only Chinese patients were enrolled in this study.

### 2.2 Ethical statement

The Ethics Committee of Shanghai Chest Hospital approved this study (KS1924). All patients signed informed consent, and the authors adhered to the Declaration of Helsinki.

### 2.3 Pulmonary function test

The pulmonary function test was conducted using a Jaeger pulmonary function tester (MasterScreen Body) within 1 week before thoracic surgery. Patients were classified as having normal or abnormal ventilatory function according to the pulmonary function guidelines formulated by the Chinese Medical Association (Chinese Thoracic Society, 2014; Chinese Thoracic Society, 2015).

### 2.4 Questionnaire

The questionnaire consisted of three parts. The first part included basic clinical information, such as age, sex, and pulmonary function test results. The second part contained two clinical scales: the modified Medical Research Council (mMRC) dyspnea scale and the chronic obstructive pulmonary disease (COPD) assessment test (CAT). Patients were asked to complete the mMRC and CAT according to their breathing status before and after the thoracic operation. The third part assessed the patients’ knowledge of pulmonary rehabilitation. The patients were required to evaluate their knowledge of lung rehabilitation based on their own condition.

### 2.5 Statistics

We used SPSS 20.0 software (IBM Co., Ltd) to perform the statistical analyses. The measurement data were expressed as mean ± SD. Comparisons between groups were performed using one-way ANOVA and the t-test. The count data were analyzed using Pearson’s χ^2^ test and Fisher’s exact probability test. The correlation between postoperative complications and pulmonary function indexes was analyzed by logistic regression. If p < 0.05, the difference was considered statistically significant.

## 3 Results

A total of 93 patients were enrolled in this study. More patients were female (58/93, 62.4%), ≤60 years old (53/93, 57.0%), and had normal pulmonary function (78/93, 83.9%). Most patients had non-small-cell lung cancer (84/93, 90.3%). The mean pre- and post-operative CAT scores were 11.35 and 14.98, respectively. The mean pre- and post-operative mMRC scores were 1.19 and 1.95, respectively. The differences in pre- and post-operative CAT and mMRC scores were 3.62 and 0.75, respectively. Most patients (53/93, 57.0%) did not know about pulmonary rehabilitation. Most patients who did know about it had received pulmonary rehabilitation knowledge from medical staff (including clinical doctors, nurses, etc.) and health promotional materials in the hospital (brochures, educational videos, etc.). Data about income, occupation, and education level were missing in most questionnaires (Table 1, Supplementary Figure S1).

**TABLE 1:** Patient characteristics.

Variable	Group	No.
Sex	Male	35
	Female	58
Age		59 (median)
	>60	40
	≤60	53
BMI		23.12
Pulmonary ventilatory function	Normal	78
	Abnormal	15
Segment removed		2.99
Pathology	Non-small-cell lung cancer (NSCLC)	84
	Small cell lung cancer (SCLC)	2
	Other tumors	1
	Benign disease	6
CAT preoperative		11.35
CAT postoperative		14.98
CAT difference		3.62
mMRC preoperative		1.19
mMRC postoperative		1.95
mMRC difference		0.75
Pulmonary rehabilitation	Know	40
	Unknown	53

NSCLC, non-small-cell lung cancer; SCLC, small cell lung cancer; mMRC, modified British Medical Research Council; CAT, COPD assessment test.

The differences in CAT and mMRC scores were calculated based on pre- and postoperative scores. Therefore, we first performed a collinearity analysis. After the calculation, the postoperative CAT and postoperative mMRC were excluded (VIF >10).

We explored the factors that influenced knowledge of pulmonary rehabilitation. Univariate analysis was first applied. Patients with abnormal pulmonary ventilatory function, higher preoperative CAT score, higher CAT differences, and higher mMRC differences showed a higher percentage of knowledge about pulmonary rehabilitation (p = 0.043, 0.029, 0.178, and 0.003, respectively) (Table 2).

**TABLE 2:** Univariate analysis of pulmonary rehabilitation.

Variable	Group	Pulmonary rehabilitation		p-value
		Unknown	Know	
Sex	Male	17	18	0.280
	Female	36	22	
Age	>60	21	19	0.527
	≤60	32	21	
BMI	<18	3	1	0.559
	18-24	32	28	
	>24	18	11	
Pulmonary ventilatory function	Normal	48	30	0.043
	Abnormal	5	10	
Segment removed		2.92 ± 1.79	3.08 ± 1.56	0.672
CAT preoperative		10.72 ± 2.92	12.20 ± 3.52	0.029
CAT difference		3.11 ± 4.20	4.30 ± 4.15	0.178
mMRC preoperative		1.19 ± 0.56	1.20 ± 0.46	0.917
mMRC difference		0.53 ± 0.64	1.05 ± 1.01	0.003

BMI, body mass index; mMRC, modified British Medical Research Council; CAT, COPD assessment test.

Then we conducted an ROC analysis to find the best cutoff values for lung segments removed, preoperative CAT score, CAT score difference, preoperative mMRC score, and mMRC score difference (Table 3; Supplementary Figure S2). Based on the best cutoff values, we categorized these indices as either poor or normal. Multivariate analysis was conducted based on previous findings. The number of lung segments removed, pulmonary ventilatory function, preoperative CAT score, CAT score difference, preoperative mMRC score, and mMRC score difference were included. Ultimately, only preoperative CAT score (HR = 6.076, 95% CI = 2.048–18.024, p = 0.001) and mMRC score difference (HR = 9.190, 95% CI = 2.583–32.696, p = 0.001) were factors associated with knowledge about pulmonary rehabilitation. Patients with worse CAT scores or larger mMRC differences took longer to learn about pulmonary rehabilitation (Table 4).

**TABLE 3:** Cutoff value of potential risk factors.

Item	Cutoff value	Sensitivity	Specificity	Youden index
Segment removed	2.5	0.650	0.509	1.159
CAT preoperative	12.5	0.425	0.868	1.293
CAT difference	3.5	0.575	0.755	1.330
mMRC preoperative	1.5	0.175	0.868	1.043
mMRC difference	1.5	0.375	0.925	1.300

mMRC, modified British Medical Research Council; CAT, COPD assessment test.

**TABLE 4:** Multivariate analysis of pulmonary rehabilitation.

Variables	Exp (B)	95% CI	p
Preoperative CAT score: <12.5 vs. > 12.5	6.076	2.048, 18.024	0.001
mMRC difference: <1.5 vs. > 1.5	9.190	2.583, 32.696	0.001

CAT, COPD assessment test; mMRC, modified British Medical Research Council.

## 4 Discussion

Surgery was the main treatment method for early-stage lung cancer patients. However, this procedure can cause many surgery-related symptoms, such as pain, fatigue, dyspnea, depression, and anxiety. These symptoms can decrease the health-related quality of life (Lowery et al., 2014). A meta-analysis revealed that the supportive care needs of people living with lung cancer include psychological/emotional, spiritual/existential, informational, patient-clinician communication, social and family-related, and cognitive aspects (Roma et al., 2013). A survey of 15 patients found several predictors that could affect the pulmonary function: lack of professional knowledge acquisition, lack of skills in symptom management, lack of emotional self-adjustment, dependence on and concerns about family support, and limited access to peer support (Ni et al., 2019). In Sharon L. Sanders’s study, lung cancer patients also expressed their requirement for additional information about their disease and treatment, exercise-related information and support (54.3%), and assistance dealing with fatigue (Sanders et al., 2010). Pulmonary rehabilitation played an important role in shortening the length of hospital stay, preserving pulmonary function, and decreasing emotional problems (depression and anxiety) (Mao et al., 2021; Chen et al., 2022; Cruz Mosquera et al., 2024; Guo et al., 2025). Those studies mainly focused on the interaction between patients and medical service providers or the patients’ close family members because symptoms might be the driving force behind the pursuit of pulmonary rehabilitation among resected lung cancer patients. Thus, we focused on the relationship between symptoms and the demand for pulmonary rehabilitation. To the best of our knowledge, this work is the first study to explore the knowledge of pulmonary rehabilitation among thoracic surgery patients.

Dyspnea was one of the main symptoms after lung cancer resection. Many reasons may account for it. First, the surgical operation removes part of the lung, thus impairing pulmonary function. Second, many patients may have lung cancer and chronic airway disease simultaneously, which means they might experience dyspnea before the surgery. Third, pain from the surgical incision or nerve injury may cause disorders of respiratory muscle movement (Moser et al., 1993; Boushy et al., 1971; Schulte et al., 2009). Because COPD coexists in many lung cancer patients, we selected the CAT and mMRC to assess the dyspnea status. Although most patients had normal pulmonary ventilatory function, some had high preoperative CAT or mMRC scores. Of course, the CAT or mMRC scores increased after surgery (Xue and Abernethy, 2010; Barğı et al., 2021). Unfortunately, most patients were not aware of pulmonary rehabilitation.

Then we explored the factors influencing cognition regarding pulmonary rehabilitation. Most clinical characteristics, including age, sex, pulmonary ventilatory function, CAT score, and mMRC score, were analyzed. In the univariate analysis, abnormal pulmonary ventilatory function, high preoperative CAT score, and higher mMRC score difference were factors associated with knowledge of pulmonary rehabilitation. However, after the multivariate analysis, only the preoperative CAT score and mMRC score difference were retained. These results were similar to those reported previously (Sohanpal et al., 2015). In Xie L’s study, severe pulmonary function impairment or a high CAT score indicated greater demand for pulmonary rehabilitation (Xie et al., 2020). A similar conclusion was reported in another cross-sectional study (Zhu et al., 2025). Several reasons may explain this: First, patients with a high CAT score before surgery tend to seek pulmonary rehabilitation before being diagnosed with lung cancer. Second, a higher mMRC score difference indicated greater pulmonary function loss due to the surgical procedure, so the patients were more likely to experience dyspnea and therefore seek additional medical assistance, including pulmonary rehabilitation (Leemans et al., 2023; Mendes et al., 2025; Bulley et al., 2009;Sahin and Naz, 2018). Those results revealed that we should not only focus on patients with poor preoperative respiratory status but also provide pulmonary rehabilitation to patients with normal CAT findings, as they may lack knowledge.

Some limitations could not be avoided. This is a single-center study, and the cohort was not large, so selection bias could not be omitted. In this manuscript, we focused on the subjective experience of patients; thus, we selected the CAT and mMRC in this research. Whether the objective index would be a better predictor of knowledge of pulmonary rehabilitation requires future studies. Some data in the questionnaire are missing, which may lead to the absence of certain important factors. In addition, the model lacks external validation. In summary, a poor preoperative CAT score and/or a sharply increasing mMRC score after surgery were the factors associated with knowledge of pulmonary rehabilitation, but assistance should also be provided to patients who do not fall into these categories, as they may lack knowledge of pulmonary rehabilitation.

## Data Availability

The raw data supporting the conclusions of this article will be made available by the authors, with legitimate reasons.
