# Supplementary material for: Operative lung cancer patients’ knowledge of pulmonary rehabilitation
Source: Front Physiol. 2026 Feb 19;17:1707620. doi: 10.3389/fphys.2026.1707620 (PMC12960077; doi:10.3389/fphys.2026.1707620)

Supplement materials:

Figure1S: The distribution of baseline characteristics of patients. mMRC: modified British medical research council; CAT: COPD assessment test.


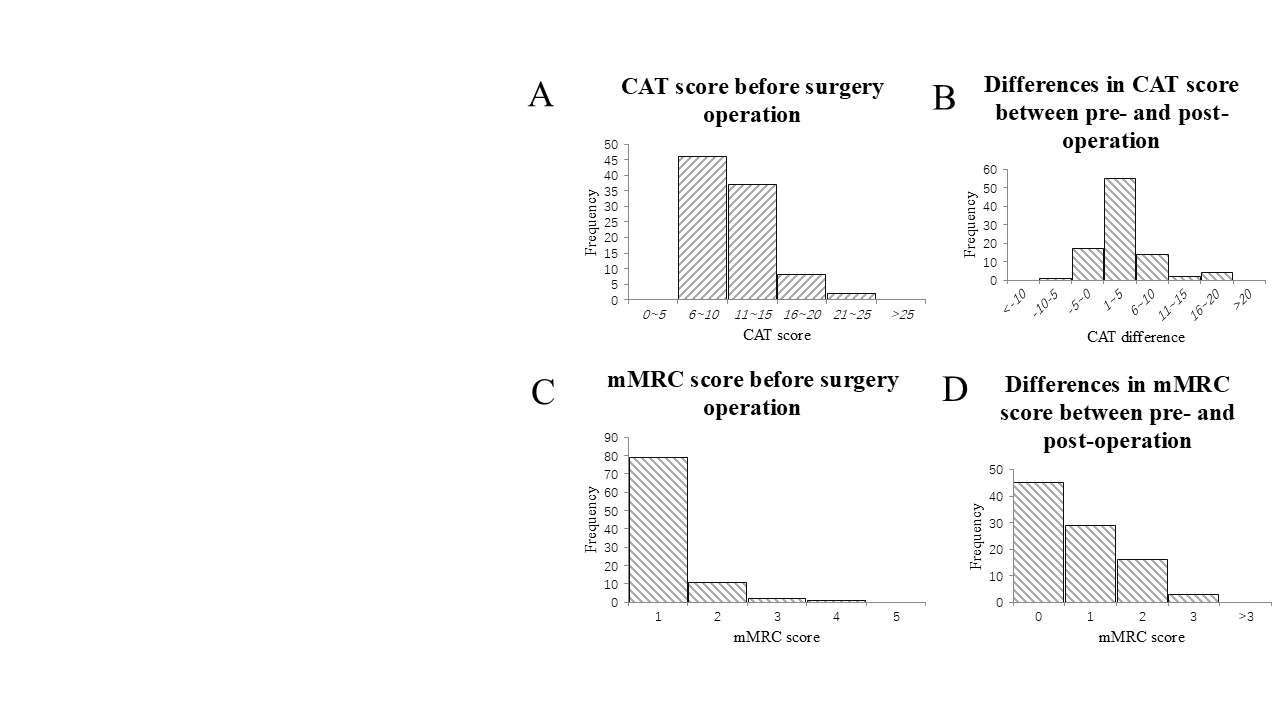


Figure S2：The ROC of index.


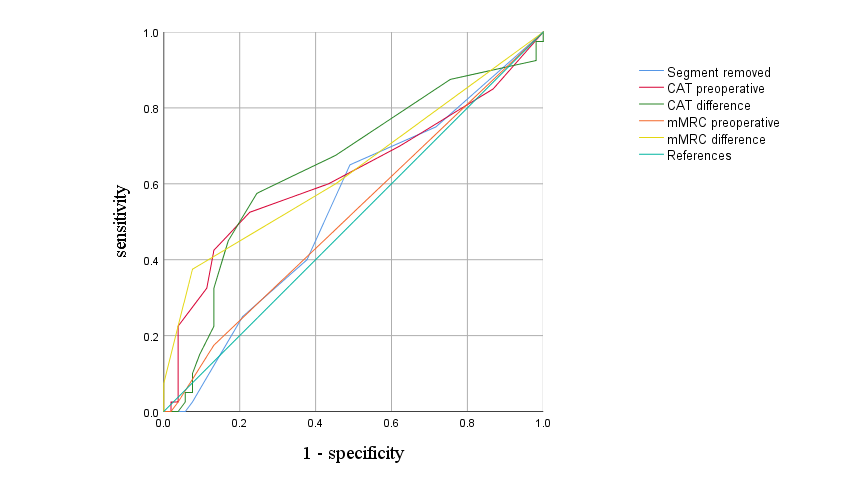

Supplement: Supplementary file 1 [file Supplementaryfile1.docx]
